# Progressive Decrease in Coronary Vascular Function Associated With Type 2 Diabetic Heart Disease

**DOI:** 10.3389/fphys.2018.00696

**Published:** 2018-06-06

**Authors:** Rajesh Katare, James T. Pearson, Jason Kar-Sheng Lew, Melanie Wei, Hirotsugu Tsuchimouchi, Cheng-Kun Du, Dong-Yun Zhan, Keiji Umetani, Mikiyasu Shirai, Daryl O. Schwenke

**Affiliations:** ^1^Department of Physiology, HeartOtago, School of Biomedical Sciences, University of Otago, Dunedin, New Zealand; ^2^Department of Cardiac Physiology, National Cerebral and Cardiovascular Center Research Institute, Suita, Japan; ^3^Bioscience Discovery Institute and Department of Physiology, Monash University, Melbourne, VIC, Australia; ^4^Japan Synchrotron Radiation Research Institute, Hyōgo, Japan; ^5^Department of Advanced Medical Research for Pulmonary Hypertension, National Cerebral and Cardiovascular Center Research Institute, Suita, Japan

**Keywords:** coronary blood flow, cardiac dysfunction, angiography, endothelial dysfunction, db/db mouse

## Abstract

**Background:** The causal factors underpinning the onset and progression of diabetic heart disease (DHD) remain to be fully elucidated. Myocardial function is critically dependent on optimal coronary blood flow. Considering vascular disease occurs early in diabetes due to endothelial dysfunction, this study aimed to determine whether impaired coronary perfusion contributes to the origins of myocardial dysfunction in DHD, or whether coronary and cardiac dysfunction are independent pathologies associated with diabetes.

**Methods:** Synchrotron radiation microangiography was used to image the coronary circulation of type-2 diabetic db/db and non-diabetic db/+ mice *in vivo* at 8, 16, and 24 weeks of age. We further assessed vascular function based on the vasodilatory responses to acetylcholine (ACh, 3 μg/kg/min), sodium nitroprusside (SNP, 5 μg/kg/min) and the Rho-kinase inhibitor, fasudil (20 mg/kg, i.v.). Cardiac function was assessed using echocardiography, and cardiac eNOS and ROCK expression were measured using immunohistochemistry.

**Results:** Coronary and cardiac function were normal in 8-week-old diabetic mice. However, by 16 weeks of age, diabetic mice had advanced cardiac dysfunction. In comparison, normal coronary perfusion was preserved in diabetes until 24 weeks of age. Moreover, only the 24-week-old diabetic mice showed clear evidence of advanced coronary vascular dysfunction, based on (i) the absence of a vasodilatory response to ACh, and (ii) an exaggerated vasodilatory response to fasudil. Interestingly, fasudil also restored normal coronary perfusion in the 24-week-old diabetic heart by restoring blood flow to previously constricted vessels (diameter < 100 μm). Importantly, there was a ubiquitous decrease, and increase, in the cardiac expression of eNOS and ROCK, respectively.

**Conclusion:** These results suggest that both cardiac and coronary dysfunction appear to have independent origins associated with diabetes and Rho-kinase pathway may be playing a role in the onset and progression of DHD.

## Background

Type II diabetes has reached epidemic proportions worldwide and is associated with numerous long-term health complications, in particular, an increased risk of developing heart disease. Considering the varying etiologies underpinning the origin of heart disease in diabetes, the term “diabetic heart disease" (DHD) is commonly used as a broad definition encapsulating heart disease, in general, in the diabetic population (Lew et al., 2017). Impaired coronary perfusion is often a pathological precursor to the onset of cardiac dysfunction in many disease states, but it is less clear if this is the case in DHD.

The functional capacity of the heart is highly dependent on adequate coronary blood flow to ensure O_2_ delivery to the myocardium is tightly matched to O_2_ demand, i.e., myocardial autoregulation (Crossman, 2004). Unlike skeletal muscle that can tolerate temporarily impaired flow, coronary flow needs to be meticulously regulated to ensure optimal oxygen and nutrient delivery to the working cardiac muscle at all times. Indeed, if coronary blood flow is adversely impaired, such as in coronary artery disease, the functional capacity of the heart can be irreversibly compromised.

Evidence in the literature suggests that impaired coronary blood flow occurs in the early stages of “*Type I*” DHD (Pearson et al., 2013). Moreover, type II diabetes is associated with progressive endothelial dysfunction (Liu et al., 2013; Lew et al., 2017; Rawal et al., 2017b), which is considered to be one of the earliest manifestations of vascular disease. To date, it remains unclear as to (i) whether the modulation of coronary perfusion is adversely impaired, or to what degree, in type II diabetes and, moreover (ii) whether impaired coronary perfusion precedes, and is therefore a causal factor, for the onset and progression of myocardial dysfunction in DHD associated with type II diabetes.

The db/db mouse has been widely used as a model of type II diabetes. The onset of diabetes in the db/db mouse is gradual, starting at 6 weeks of age and characterized by an obese phenotype with hyperinsulinemia and subsequent hyperglycemia as a result of two mutant copies of the leptin receptor gene (Sennott et al., 2008). We along with others have demonstrated that db/db mouse closely reflects the changes occurring in the type 2 human diabetic heart (Aasum et al., 2003; Rawal et al., 2017a,b).

In this study, we aimed to establish whether coronary vascular dysfunction, and thus impaired coronary blood flow, acts to facilitate the onset of myocardial dysfunction of the heart in the db/db mouse model of type II diabetes.

## Research Design and Methods

### Animals

This study was carried out in accordance with the guidelines of the Physiological Society of Japan and the ARRIVE guidelines. The protocol was approved by the Animal Ethics Committee of SPring-8. Experiments were conducted on male db/db diabetic mice and non-diabetic mice aged 8 weeks old (*n* = 8 and *n* = 7, respectively), 16 weeks old (*n* = 10 and *n* = 9, respectively), and 24 weeks old (*n* = 8 and *n* = 7, respectively). All mice were on a 12 h light/dark cycle at 25 ± 1°C and provided with food and water *ad libitum*.

### Echocardiography

One day prior to angiographic imaging of the coronary circulation, functional parameters and dimensions of the left ventricle were measured in all mice using a Vevo 20 MHz Micro-Scan 250 transducer and a Vevo2100 Imaging Platform (VisualSonics, Toronto, ON, Canada). Mice were anesthetized with isoflurane/oxygen (1.5–5%/1L.min^-1^) and placed supine on an imaging stage equipped with a warming pad for maintaining body temperature at 37°C. Standard B mode (2D) images of the heart and pulsed Doppler images of the mitral valve inflow (to estimate the E/A ratio and deceleration time) were acquired. Left ventricular ejection fraction (EF), fractional shortening (FS), end systolic volume (ESV), and end diastolic volume (EDV) were determined in M mode as described previously (Katare et al., 2011). All measurements and calculations were averaged from three separate measurements according to the American Society of Echocardiography guidelines. Data analysis was performed offline with the Vevo^®^ LAB desktop Software (v1.7.1, VisualSonics, Toronto, ON, Canada).

### Glycated Hemoglobin

Fifty microliters of blood was collected from a small tail snip when mice were anesthetized for echocardiography and HbA1c level was determined using a Bio-Rad variant II turbo system (Bio-Rad Laboratories, Hercules, CA, United States).

### Synchrotron Radiation Coronary Microangiography

The coronary circulation was visualized, *in vivo*, using synchrotron radiation (SR) microangiography at the Super Photon ring-8 GeV (SPring-8) facility, BL28B2 beam line, Hyogo, Japan as previously described (Pearson et al., 2013; Shirai et al., 2014). Mice were anesthetized with 2,2,2-tribromoethanol (Avertin^®^, 0.3 gm/kg, i.p). Body temperature was maintained at 37°C using a thermostatically controlled heating pad. The trachea was cannulated and the mouse mechanically ventilated (MouseEvent^®^ PhysioSuite, Kent Scientific Corporation, Torrington, CT, United States). The jugular vein was cannulated for fluid/drug administration. The right common carotid artery was cannulated with a fine PE10 catheter that was advanced down the carotid artery in close proximity to the aortic valve, so that iodinated contrast medium (Iomeron 350, Bracco-Eisai Co., Ltd., Tokyo) could be injected directly into the coronary vessels using a Harvard 2000 syringe pump. The carotid catheter was also used to intermittently measure arterial blood pressure (ABP).

**FIGURE 1:**
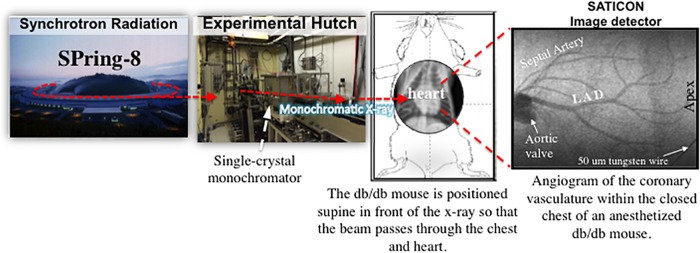
A schematic diagram showing the experimental set-up for coronary microangiography in an anesthetized mouse model using monochromatic synchrotron radiation at the SPring-8 facilities in Hyogo, Japan (modified from Pearson et al., 2018).

The surgically prepared mouse was strategically positioned supine in front of, and perpendicular to, the SATICON X-ray detector (Hitachi Denshi Techno-system, Ltd., Tokyo, Japan and Hamamatsu Photonics, Shizuoka, Japan) so that the thorax was in alignment with a 9.5 mm × 9.5 mm imaging field (**Figure 1**). During each brief imaging scan, monochromatic SR at 33.2 keV at a flux of ∼10^10^ photons/mm^2^/s passed through the chest of the anesthetized mouse. A syringe pump (a Harvard PHD200 pump, Harvard Apparatus, Holliston, MA, United States) was used to inject a single bolus of contrast agent (Iomeron 350; Eisai Co., Ltd., Tokyo, Japan) at high-speed (0.2 ml @ 0.4 ml/s) intra-arterially into the coronary circulation and cine-radiograms were obtained over 2 s. For each 2-s period of scanning (a single exposure sequence), 100 frames were recorded (10 bit resolution) with a shutter open time of ∼1 ms. Mice were given at least 10 min to recover from each bolus injection of contrast agent.

A baseline image of the coronary circulation was first collected to assess any differences in the vessel branching network between the diabetic and non-diabetic mice of various ages. Following baseline imaging, coronary angiograms were also recorded in response to: (i) acetylcholine (ACh, 10.0 μg/kg/min for 5 min, i.v.) to assess endothelium-dependent vasodilation, (ii) the NO donor sodium nitroprusside (SNP, 10 μg/kg/min for 5 min, i.v.) to assess endothelium-independent vasodilation, and (iii) fasudil (20 mg/kg, i.v.), a Rho-kinase inhibitor. Microangiography was performed after the 5th min of ACh and SNP infusion, and 15 min following the bolus dose of fasudil. At least 10–15 min was required for all cardiovascular variables to return to baseline values following ACh and SNP drug interventions.

### Data Analysis

Arterial blood pressure was recorded and data sampled at 400 Hz using a PowerLab data-acquisition system (model 8/S, ADInstruments Ltd., New Zealand). Heart rate (HR) was derived from the arterial systolic peaks. The imaging analysis program Image Pro-plus (ver. 7.0.1, Media Cybernetics, Rockville, MD, United States) was used to enhance contrast and the clarity of angiogram images as previously described (Shirai et al., 2014). Angiogram images were randomly coded so that the manual counting of vessel branches could be conducted in a blinded manner. The diameters of 2–4 vessels of each branching generation were measured in each mouse to ensure a wide variety of vessel sizes were selected from each frame. Vessels were categorized according to internal diameter (ID, μm); 50–100 μm, 100–150 μm, 150–200 μm, and 200–300 μm. A 50 μm-thick tungsten filament, placed directly across the corner of the detector’s window, appearing in all recorded images and was subsequently used as a reference for calculating vessel ID.

### Immunohistochemistry

To determine the possible mechanisms underlying the coronary dysfunction in the diabetic heart we analyzed the expression of RhoA/Rho Kinase signaling pathway which has been shown to be ubiquitously up-regulated throughout the vasculature, myocardium, and extracellular tissue of the diabetic heart (Mahavadi et al., 2017), and thus may also be directly involved in the impairment of both myocardial and coronary function (Zhou et al., 2011; Soliman et al., 2012; Waddingham et al., 2015a). Myocardial tissue collected from 8-, 16-, and 24-week-old mice were fixed in 4% formalin and cryosectioned for immunohistochemistry analysis using previously published protocols (Pearson et al., 2013). In brief, five-micron cryosections were washed with PBS thrice after which the sections were boiled for 30 min in citrate buffer (pH6.0) for antigen retrieval. After serial washing with PBS (pH 7.4), non-specific protein binding was blocked with 20% normal goat serum (Dako, Glostrup, Denmark) for 30 min. The sections were then incubated with the primary antibody against ROCK1 (1:200; #ab45171, Abcam, Cambridge, MA, United States) or eNOS (1:100, Life Technologies, New Zealand) overnight at 4°C. Sections were then incubated with goat anti-rabbit horseradish peroxidase (HRP) (Dako, Glostrup, Denmark) for 40–60 min at room temperature and developed using diaminobenzidine (Vector Laboratories, Inc., Burlingame, CA, United States). The sections were then counter-stained with hematoxylin, dehydrated, cleared and mounted with DPX mountant. Images were captured using an automatic serial scanner (Aperio, Leica). Staining intensity was then analyzed using ImageJ and expressed as mean intensity per section.

### Statistical Analysis

All statistical analyses were conducted using Graphpad Prism (v7.0b, Graphpad Software Inc.). All results are presented as either means ± standard error of the mean (SEM), or illustrated as a box-and-whisker plot. Given that the variance of angiography data was similar between groups, two-way ANOVAs (repeated measures) were used to test whether vessel caliber (ID), vessel number, and hemodynamic responses differed significantly between mice of different ages, diabetic status, and their interaction. One-way ANOVA (factorial) was used to test for differences (i) within groups between baseline and drug administration and (ii) between groups for each drug treatment. Where statistical significance was reached, *post hoc* analyses were incorporated using the paired, or unpaired *t*-test with the Dunnett’s correction (temporal effects) or Sidak’s correction (between groups) for multiple comparisons. A *P*-value ≤ 0.05 was predetermined as the level of significance for all statistical analyses.

## Results

### Diabetes Induced Deterioration of Cardiac Function in db/db Mice

The structural and functional capacity of the heart, including coronary perfusion, of 8-week old db/db diabetic mice was identical to the 8-week non-diabetic counterpart (control), indicating that at 8 weeks of age, diabetic mice had normal cardiac function (**Table 1**). Hyperglycemia was confirmed in the 8-week old db/db ‘*diabetic*’ mice with HbA1c levels exceeding the upper detectable limit (>14%), compared to the level of 4.8 ± 0.1% in non-diabetic mice (**Table 1**).

**Table 1:** Measures of cardiac and haemodynamic function in non-diabetic (ND) and diabetic (Diab) db/db mice at 8, 16, and 24 weeks of age.

	8 weeks		16 weeks		24 weeks	
	ND (*n* = 7)	Diab (*n* = 8)	ND (*n* = 9)	Diab (*n* = 10)	ND (*n* = 7)	Diab (*n* = 8)
BW (g)	26.6 ± 0.2	42.8 ± 0.8^∗^	29.4 ± 0.7	54.0 ± 1.9^∗^	31.3 ± 0.6	54.7 ± 2.7^∗^
HbA1c (%)	4.8 ± 0.1	>14^∗^	4.7 ± 0.1	>14^∗^	4.4 ± 0.1	12.3 ± 0.7^∗^
-[mmol/mol]	[29.3 ± 1.3]	[>130^∗^]	[28.0 ± 1.0]	[>130^∗^]	[25.0 ± 1.0]	[110.7 ± 8.6^∗^]
EF (%)	70 ± 2	73 ± 1	71 ± 3	50 ± 2^∗†^	74 ± 2	51 ± 2^∗†^
FS (%)	39 ± 2	41 ± 1	40 ± 3	25 ± 2^∗†^	43 ± 2	26 ± 1^∗†^
EDV (μl)	58 ± 4	63 ± 4	74 ± 3	81 ± 3^†^	68 ± 2	69 ± 5
ESV (μl)	17.1 ± 1.1	17.0 ± 1.5	21.6 ± 2.8	40.9 ± 3.4^∗†^	15.8 ± 2.1	34.5 ± 3.7^∗†^
E/A ratio	1.81 ± 0.01	1.82 ± 0.02	1.78 ± 0.01	2.25 ± 0.10^∗†^	1.74 ± 0.02	2.17 ± 0.04^∗†^
Decl time (ms)	32 ± 0.84	32.52 ± 0.61	32.78 ± 0.32	23.05 ± 0.97^∗†^	31.93 ± 0.84	23.68 ± 1.01^∗†^
ABP (mmHg)	70 ± 6	80 ± 8	73 ± 8	69 ± 4	73 ± 7	90 ± 8
HR (.min^-1^)	486 ± 16	488 ± 20	437 ± 28	404 ± 12	458 ± 27	404 ± 28

*Data are presented as mean ± SEM. EF, ejection fraction; FS, fractional shortening; EDV, end-diastolic volume; ESV, end-systolic volume; Decl time, deceleration time; ABP, arterial blood pressure; HR, heart rate. ^∗^Significant difference between non-diabetic and diabetic mice (^∗^P < 0.01). ^†^Significantly different to “8 weeks” (^†^P < 0.01). There was no significant difference between “16 weeks” and “24 weeks” for all measured variables*.

By 16 weeks of age diabetic mice had clear signs of systolic dysfunction (e.g., EF = 50 ± 2%, *cf.* 71 ± 3% in non-diabetics; *P* < 0.01) and diastolic dysfunction (increased E/A ratio; 2.25 ± 0.1, *cf.* 1.78 ± 0.01 in non-diabetics; *P* < 0.01 and reduced deceleration time; 23.05 ± 0.97, *cf.* 32.78 ± 0.32 in non-diabetics) (**Table 1**). The severity of cardiac dysfunction did not significantly worsen from 16 to 24 weeks of age in diabetic mice (**Table 1**).

### Synchrotron Microangiography Imaging of the Coronary Circulation

**FIGURE 2:**
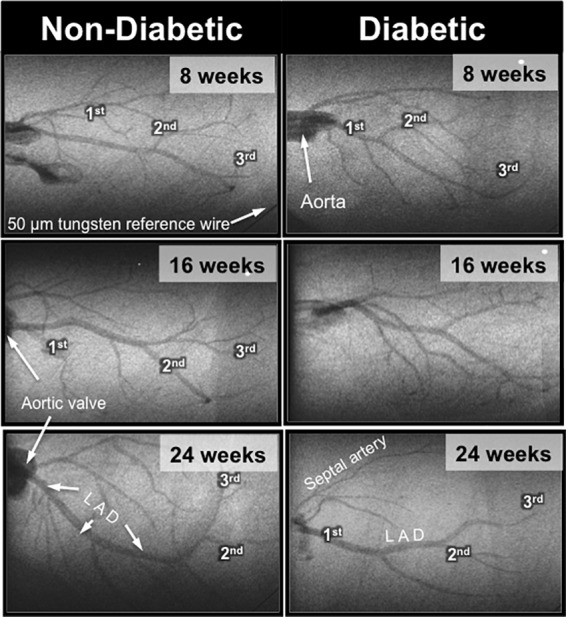
Representative microangiogram images showing the contrast-enhanced coronary arterial branching network down to the 3rd order in anesthetized db/db non-diabetic **(Left)** and db/db diabetic **(Right)** mice at 8, 16, and 24 weeks of age. The tungsten wire in the bottom right corner of both angiogram frames is a reference of 50 μm diameter.

**FIGURE 3:**
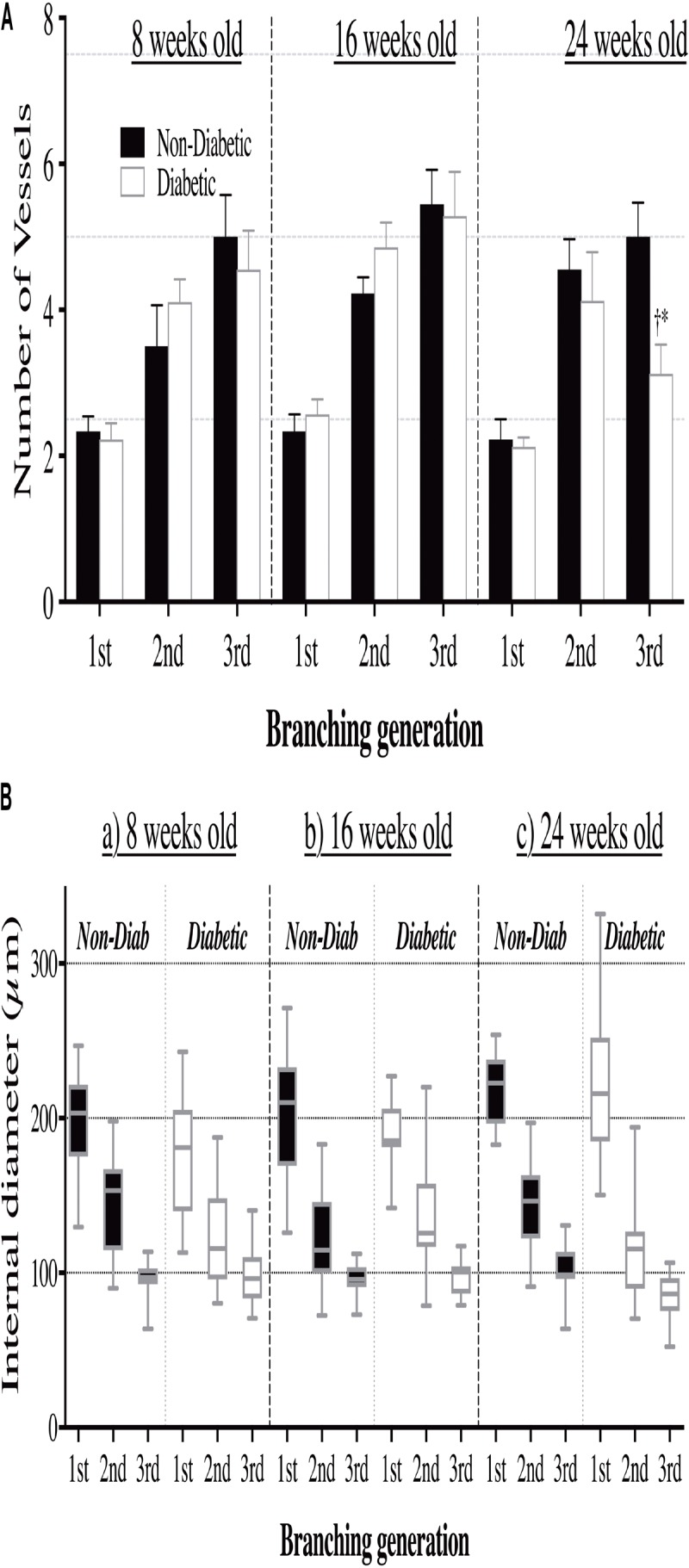
**(A)** The number of opaque vessels (mean ± SEM) and **(B)** the range of vessel sizes (box and whisker graph), at each of the first three branching generations of the coronary circulation in anesthetized db/db diabetic and non-diabetic mice at 8 weeks old (*n* = 8 and *n* = 7, respectively), 16 weeks old (*n* = 10 and *n* = 9, respectively), and 24 weeks old (*n* = 8 and *n* = 7, respectively). Quantitative data presented in **A** show a decrease in the number of 3rd order branches in the 24-week-old diabetic mice compared to the (i) 8 week old diabetic mice (^∗^*P* < 0.05) and (ii) age-matched non-diabetic animals (^†^*P* < 0.05).

Interestingly, the impaired cardiac function in 16-week-old diabetic mice was not matched with an adverse change in coronary blood flow distribution (i.e., perfusion). Indeed, angiographic evidence (**Figure 2**) revealed that the number and size of 1st, 2nd, and 3rd order coronary vessel branches did not significantly differ from the 16-week non-diabetic (control) mice (**Figures 3A**,**B**). However, by 24 weeks of age, coronary perfusion had significantly declined, evident in that there were fewer radiopaque vessels of the 3rd branching generation (within the field-of-view) for diabetic mice (3.1 ± 0.4 vessels; *n* = 8) compared to non diabetic counterparts (5.0 ± 0.5 vessels, *P* < 0.05; *n* = 9) (**Figures 2**,**3A**).

### Impaired Endothelium-Dependent Vasodilation in Diabetic Mice

**FIGURE 4:**
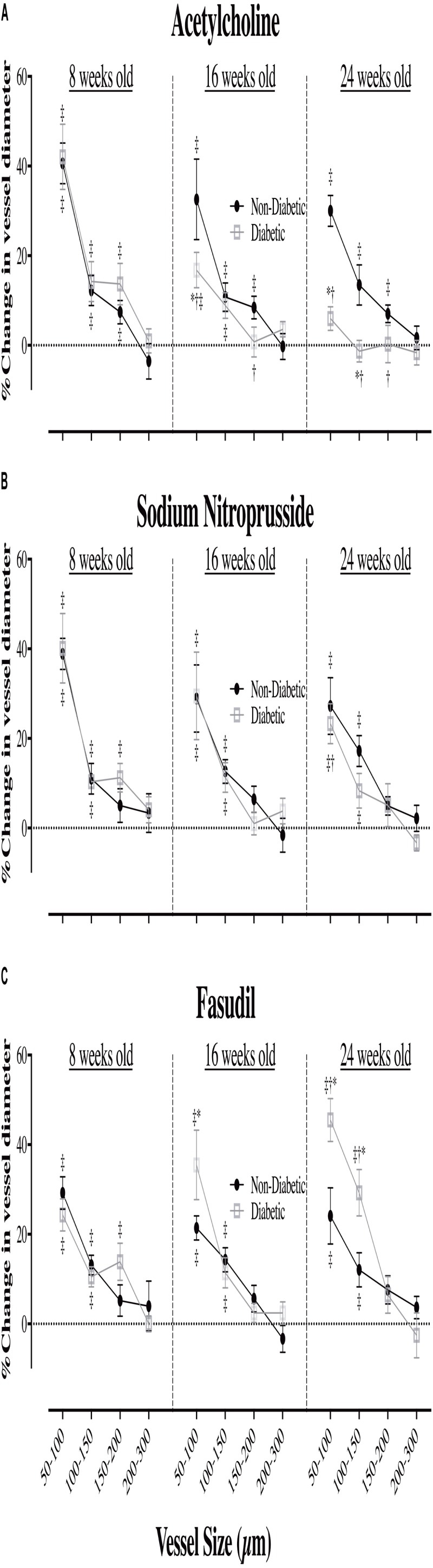
The relationship between coronary vessel size and the magnitude (%) of change in vessel internal diameter (ID) in anesthetized db/db diabetic and non-diabetic mice (mean ± SEM) at 8 weeks (*n* = 8 and *n* = 7, respectively), 16 weeks (*n* = 10 and *n* = 9, respectively), and 24 weeks of age (*n* = 8 and *n* = 7, respectively) in response to **(A)** acetylcholine (ACh – 10.0 μg/kg/min for 5 min), **(B)** sodium nitroprusside (SNP – 10.0 μg/kg/min for 5 min), and **(C)** fasudil (20 mg/kg, i.v.). ^∗^Significant difference between non-diabetic vs. diabetic (*P* < 0.05). ^†^Significantly different from 8-week old mice (*P* < 0.05). ^‡^Significant reduction/increase in vessel caliber, compared to baseline (*P* < 0.05).

**FIGURE 5:**
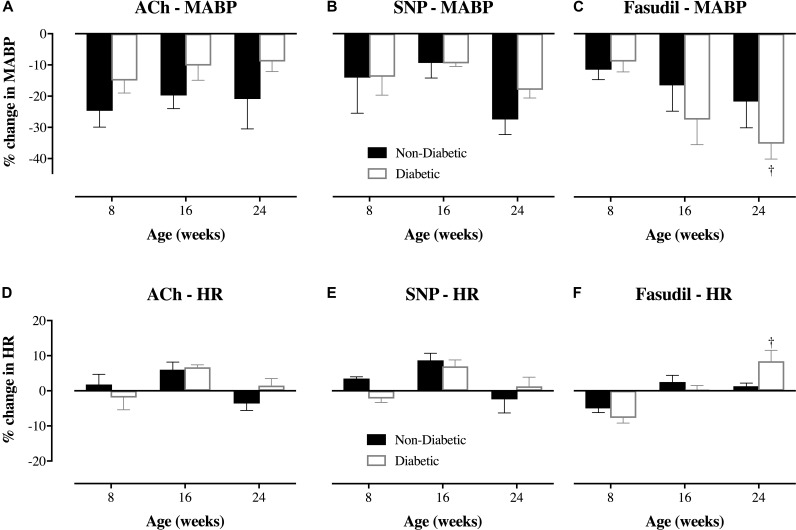
Magnitude of change (%) in mean arterial blood pressure (MABP) and heart rate (HR) of db/db diabetic and non-diabetic mice (mean ± SEM) at 8 weeks old (*n* = 8 and *n* = 7, respectively), 16 weeks old (*n* = 10 and *n* = 9, respectively), and 24 weeks old (*n* = 8 and *n* = 7, respectively) in response to **(A,D)** acetylcholine (ACh – 10.0 μg/kg/min for 5 min), **(B,E)** sodium nitroprusside (SNP – 10.0 μg/kg/min for 5 min), and **(C,F)** fasudil (20 mg/kg, i.v.). ^†^Significantly different from the response of 8-week-old diabetic mice (*P* < 0.05).

In the 8-week old db/db mice, ACh (10.0 μg/kg/min for 5 min, i.v.) caused extensive coronary vasodilation of all vessels with an ID < 200 μm, which was similar for both diabetic and non-diabetic mice (e.g., ∼40% increase in ID for the 50–100 μm vessels; *P* < 0.01) (**Figure 4A**). However, as the diabetic mice aged, the dilatory responses to ACh progressively declined with the magnitude of vasodilation for the 50–100 μm microvessels blunted (*P* < 0.05) at 16 weeks of age (17% increase in ID) and completely abolished by 24 weeks of age (**Figure 4A**). For both diabetic and non-diabetic mice, ACh did not significantly alter heart rate (HR), but it did cause a significant decrease in Mean arterial blood pressure (MABP, 20.9 and 8.9% decrease in MABP, respectively), which trended to be smaller in magnitude, albeit not significant, for the diabetic mice (**Figures 5A,D**). The HR and MABP responses to ACh did not change with age (**Figure 5A**).

**FIGURE 6:**
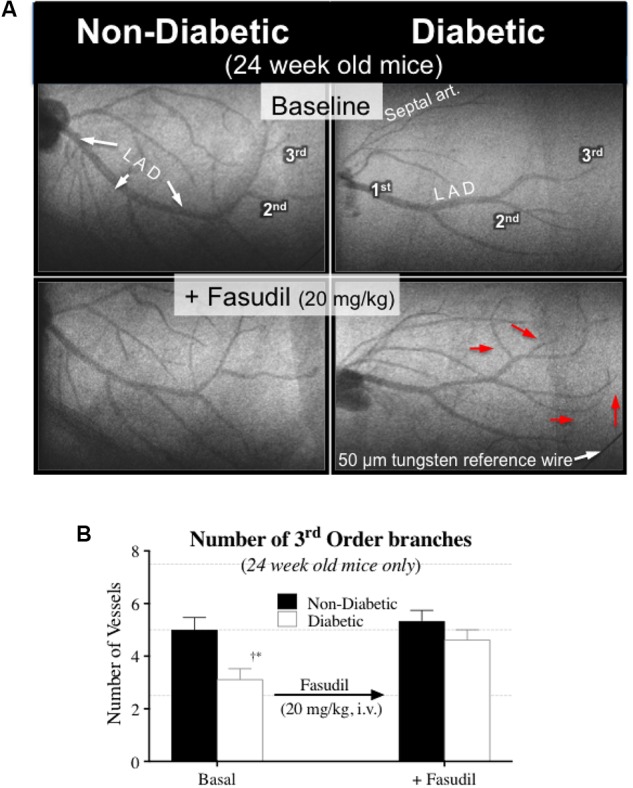
**(A)** Microangiogram images showing the effect of fasudil (20 mg/kg, i.v.) in improving coronary blood flow distribution in 24-week-old diabetic mice, via the recruitment of additional 3rd order branches (red arrows). **(B)** Quantitative data (mean ± SEM) of the coronary angiograms showing an increase in the number of 3rd order branches in the 24-week-old diabetic mice (*n* = 8) following fasudil administration. ^∗^Significant difference between diabetic and non-diabetic mice (*P* < 0.05). ^†^Significantly different from 8 weeks of age (*P* < 0.05).

### Endothelium-Independent Vasodilation Is Preserved in Diabetes

The administration of exogenous NO (SNP at 10 μg/kg/min for 5 min), caused significant coronary vasodilation of all vessels ID < 150 μm, which was of similar magnitude for both diabetic and non-diabetic mice of all ages (**Figure 4B**). Moreover, the vasodilatory responses to SNP did not significantly change with age; with the one exception that the magnitude of dilation for the 50–100 μm coronary vessels in diabetic mice was attenuated from 8 to 24 weeks of age (increase in ID of 40% down to 23%, respectively; *P* = 0.04). SNP did not significantly alter HR, but it did induce a significant decrease in MABP of similar magnitude for both diabetic and non-diabetic mice, which did not significantly change with age (**Figures 5B,E**).

### Increased Rho-Kinase; A Possible Mechanism for Sustained Vasoconstriction in Advanced Diabetes

The Rho-kinase inhibitor, fasudil (20 mg/kg), caused coronary vasodilation of all vessels ID < 150 μm, to a similar extent for both 8-week-old diabetic and non-diabetic mice (**Figure 4C**). The response to fasudil did not change with age in non-diabetic mice. Interestingly, diabetic mice exhibited a progressive and significant increase in the vasodilatory response to fasudil, so that by 24 weeks of age the magnitude of dilation for the 100–150 μm vessels was almost double that observed at 8 weeks of age (increase in microvessel ID of 45 ± 5% *cf.* 24 ± 4%, respectively; *P* < 0.01) (**Figure 4C**). In addition, in the 24-week-old diabetic mice only, fasudil also promoted the recruitment and (re)perfusion of vessels of the 3rd order branching that were not previously visibly opaque in baseline angiographs, thereby abolishing the difference in the number of 3rd generation branches between 24-week old diabetic vs. non-diabetic mice (4.6 ± 0.4 vs. 5.3 ± 0.4 vessels, respectively) (**Figure 6**). Fasudil caused a decrease in systemic ABP in non-diabetic mice that did not change with age (**Figure 5C**). In contrast, in the diabetic mice, the fasudil-mediated hypotension was significantly augmented with age (*P* < 0.05) in diabetic mice (9, 27, and 35% decrease in MABP for 8-, 16-, and 24-week-old mice, respectively) (**Figure 5C**), which was associated with an increase in HR that was significantly only for the 24-week-old mice (8% increase in HR; **Figure 5F**).

### Elevated Expression of Rho-Kinase Protein (ROCK1) and Reduced Expression of eNOS Protein in the Diabetic Heart

**FIGURE 7:**
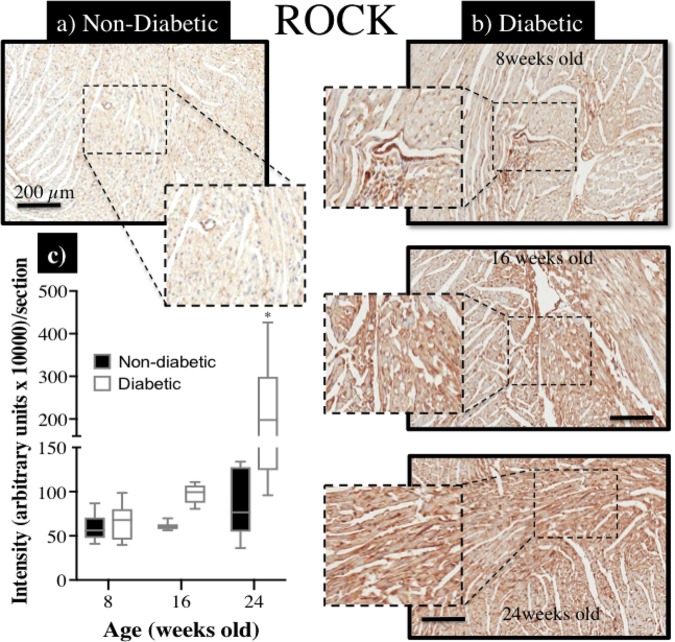
Immunohistochemical assessment of Rho-kinase protein (ROCK1) expression within myocardial sections retrieved from db/db **(a)** non-diabetic mice and, **(b)** diabetic mice at 8, 16, and 24 weeks of age; **(c)** the intensity of ROCK staining (brown precipitate) appeared to be progressively enhanced with age in diabetic hearts, compared to non-diabetic hearts, and significantly more so by 24 weeks of age (^∗^*P* < 0.05).

**FIGURE 8:**
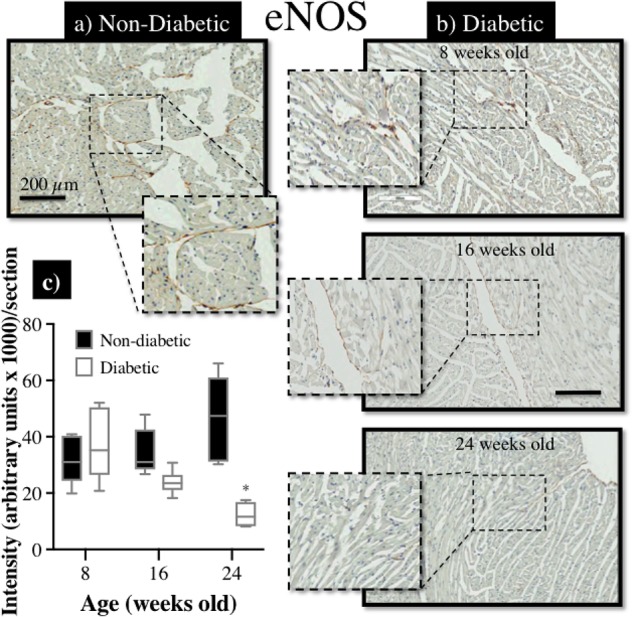
Immunohistochemical assessment of eNOS protein expression in myocardial sections retrieved from db/db **(a)** non-diabetic mice and, **(b)** diabetic mice at 8, 16, and 24 weeks of age; **(c)** the intensity of eNOS staining (brown precipitate) appeared to be progressively reduced with age in diabetic hearts, compared to non-diabetic hearts, and significantly more so by 24 weeks of age (^∗^*P* < 0.05).

Immunohistochemical staining of sections from diabetic and non-diabetic hearts showed that there was a ubiquitous increase in the expression of ROCK1 protein throughout the diabetic heart (**Figures 7a,b**), which began from 16 weeks of age (NS), and was significantly elevated by 24 weeks of age (**Figure 7c**). In contrast, there was a progressive decrease in the expression of total eNOS protein in the diabetic heart (**Figures 8a,b**), although the difference, compared to non-diabetic mice, was once again not statistically significant until 24 weeks of age (**Figure 8c**).

## Discussion

The primary findings of this study highlight that (i) impaired coronary perfusion and endothelial dysfunction only become fully established in the db/db mouse model of diabetes at 24 weeks of age, although moderate impairment of endothelium dependent dilation is evident in the microvessels at 16 weeks of age, and (ii) sustained ROCK-mediated vasoconstriction may potentially have a role in reduced coronary perfusion in type 2 diabetes at an advanced stage. Importantly, cardiac dysfunction appears to develop concurrently with coronary dysfunction, as a direct result of diabetes, rather than as a causal result of the progressive coronary dysfunction.

It is generally accepted that the key hallmarks of diabetes, such as hyperglycemia, dyslipidemia, and insulin resistance collectively contribute to the early pathophysiological molecular, structural, and myocardial abnormalities (Wright et al., 2009; Cook et al., 2010; Bradley et al., 2016), which underpin DHD. Indeed, diabetes itself dramatically increases the risk of heart disease (Kannel and McGee, 1979). Importantly, one of the key determinants of cardiovascular outcome is the duration with which a patient has diabetes (Fox et al., 2004).

In this study, we aimed to track the progressive changes in coronary perfusion from the early stages of diabetes in a db/db mouse model (8 weeks old) to the later stages (24 weeks) when severe cardiac dysfunction had become well advanced, and determine whether impaired coronary perfusion coincides with the onset of cardiac dysfunction; at least in the db/db mouse. The db/db mouse exhibits striking similarities to the disease progression in humans, and previous studies have shown that db/db mice develop numerous diabetes-induced cardiac complications, such as diastolic and systolic dysfunction, microangiopathy, fibrotic remodeling, and eventually progressive loss of cardiac cells, making them an ideal model to investigate the effect of diabetes on coronary perfusion (Fuentes-Antras et al., 2015).

Diabetes is a major cause of ischemic coronary artery disease (Naito and Miyauchi, 2017) and coronary endothelial dysfunction (Matsunaga et al., 1996; Di Carli et al., 1999). Although the functional capacity of the heart is highly dependent on the preservation of adequate coronary perfusion, we noted in this study that impaired coronary perfusion was not a ‘pre-cursor’ to the development of cardiac dysfunction. Rather, both coronary and cardiac dysfunction appeared to develop concurrently as a direct result of the diabetes, at least by 16 weeks of age. One limitation of this study is that we did not identify the actual time point at which cardiac dysfunction started to appear, between 8 and 16 weeks of age.

Although the mechanisms underpinning the onset of cardiac dysfunction are likely to be multifactorial, emerging evidence in the literature implicates early molecular alterations in the diabetic heart as precursors for facilitating the structural and functional defects that manifest in the later stages of cardiac disease (Waddingham et al., 2015b; Mizamtsidi et al., 2016). In particular, we have recently reported that down-regulation of pro-angiogenic microRNAs (miR)-126 and -132 in the db/db mouse heart as early as 8 weeks of age, appears to be a primary catalyst for the inevitable structural decrease in coronary capillary and arteriole density evident at 20 weeks of age (Rawal et al., 2017b). Moreover, early down-regulation of anti-fibrotic miR-15a and -15b in the myocardium of 12 week-old db/db mice facilitates adverse myocardial fibrosis that is only evident by 20 weeks of age (Rawal et al., 2017a). Although we did not measure the expression of miRs in this study, it is likely that early dysregulation in proangiogenic and other yet to be identified miRs could underpin coronary artery and cardiac dysfunction in this study.

### Endothelial Modulation of Vascular Tone

The endothelium plays an important role in modulating coronary vascular tone through the release of vasoactive mediators such as NO, ET-1, prostacyclin, serotonin, and thromboxane. Not surprisingly, therefore, endothelial dysfunction has been implicated as an important contributor to coronary arterial dysfunction in diabetes (Koltai et al., 1997; Tawfik et al., 2006), which is consistent with the blunted response to ACh and reduced expression of eNOS as observed in this study. Nonetheless, it must be acknowledged that we did not specifically determine if eNOS activity or nitric oxide bioavailability in this study. The many causes of endothelial dysfunction have been linked to key hallmarks of diabetes such as oxidative stress (Tawfik et al., 2006; Idris-Khodja et al., 2016), insulin resistance, ROCK activation, and proinflammatory signaling of Wnt5a (Breton-Romero et al., 2016).

In spite of the development of coronary endothelial dysfunction in the diabetic mice of this study, the sensitivity of the vascular smooth muscle to NO was largely preserved, as previously reported (Koltai et al., 1997). A key observation in this study was that, by 24 weeks of age, coronary perfusion was reduced in diabetic mice, based on a reduction in the number of 3rd order vessels. And although exogenous NO donation (SNP) effectively dilated already-perfused vessels, it was not able to restore myocardial perfusion.

### Rho-Kinase Mediated Vasoconstriction in Type 2 Diabetes

Perhaps one of the most fundamental findings of this study is that acute Rho-kinase inhibition (using fasudil) restored coronary blood flow distribution in 24-week-old diabetic mice, not only by the widespread dilatation of already-perfused vessels (ID > 100 μm), but also by restoring blood flow to vessels that were not previously visibly opaque, i.e., constricted below limits of detection (ID < 50 μm). Moreover, fasudil triggered a [reflex] increase in heart rate in the 24-week-old diabetic mice, thereby increasing metabolic demand of the heart, which itself may have been a contributing local factor to the observed coronary dilation.

Hyperglycemia is an important factor promoting the accumulation of reactive oxygen species (ROS) (Fiorentino et al., 2013), and it is this adverse oxidative stress that underpins, not only endothelial dysfunction as noted above, but also vascular smooth muscle proliferation, apoptosis and, most notably, upregulation of the RhoA/Rho Kinase pathway (Mahavadi et al., 2017). Importantly, RhoA/Rho kinase activation has been implicated as a leading cause of several different etiologies of myocardial dysfunction (Zhou et al., 2011; Waddingham et al., 2015a; Li et al., 2016; Lai et al., 2017) as well as coronary microvascular dysfunction (Shiroto et al., 2009; Aizawa et al., 2012; Pearson et al., 2013). In this study we identified a progressive and ubiquitous over-expression of ROCK1 throughout the Type 2 diabetic heart that may explain the augmented vasodilatory (and reperfusion) response to fasudil in the 24-week-old db/db mice. However, further research is now essential to identify the specific mechanisms that underpin the increased ROCK expression/activation and its preferential localization within cardiomyocytes as well as coronary endothelial and/or smooth muscle cells. Ultimately, chronic ROCK inhibition as a potential therapy for both cardiac and coronary dysfunction in diabetes warrants future exploration.

We have previously described the high definition achieved with SR microangiography for visualizing and imaging microvessels of various vascular beds (Shirai et al., 2013), including the coronary circulation (Pearson et al., 2018). One significant limitation with SR is that it is not possible to assess (i) the integrity of the vascular wall for smooth muscle hypertrophy, which would reduce flow, and (ii) changes in the expression of key proteins that regulate vascular tone, such as ROCK and eNOS. Indeed, SR is only able to measure the internal diameter of perfused vessels (assuming the vessel contains sufficient contrast medium) and is therefore a simple, albeit useful, method for assessing gross anatomical changes in the coronary circulation of the diabetic heart.

## Conclusion

We have utilized SR microangiography to highlight the progressive decline in coronary perfusion and function associated with type 2 diabetes in the db/db mouse model. Impaired coronary function does not appear to precede the onset of cardiac dysfunction suggesting that the pathological hallmarks of diabetes (e.g., oxidative stress) independently evoke coronary and cardiac dysfunction. Importantly, Rho-kinase inhibition was effective at restoring normal coronary perfusion in the dysfunctional diabetic heart by dilating coronary vessels and recruiting previously non-perfused vessels. Future studies are warranted to determine the precise role of RhoA/Rho Kinase pathway in impaired coronary perfusion in diabetes.

## Author Contributions

RK and JK-SL performed immunohistochemistry experiments and RK further contributed to the editing of the manuscript. MW, HT, KU, and JP contributed to the angiography experiments, and JP with MS further contributed to the interpretation of data and editing of the manuscript first and second draft. JK-SL, C-KD, and D-YZ performed the echocardiography experiments. DS conceived, designed, and supervised the study, contributed to angiography experiments, interpreted data and was primary writer of the manuscript.

## Conflict of Interest Statement

The authors declare that the research was conducted in the absence of any commercial or financial relationships that could be construed as a potential conflict of interest.
